# N-Acetylcysteine Prevents Spatial Memory Impairment Induced by Chronic Early Postnatal Glutaric Acid and Lipopolysaccharide in Rat Pups

**DOI:** 10.1371/journal.pone.0078332

**Published:** 2013-10-24

**Authors:** Fernanda S. Rodrigues, Mauren A. Souza, Danieli V. Magni, Ana Paula O. Ferreira, Bibiana C. Mota, Andreia M. Cardoso, Mariana Paim, Léder L. Xavier, Juliano Ferreira, Maria Rosa C. Schetinger, Jaderson C. Da Costa, Luiz Fernando F. Royes, Michele R. Fighera

**Affiliations:** 1 Centro de Ciências da Saúde, Departamento de Neuropsiquiatria, Universidade Federal de Santa Maria, Santa Maria, Rio Grande do Sul, Brasil; 2 Centro de Ciências Naturais e Exatas, Programa de Pós-graduação em Ciências Biológicas: Bioquímica Toxicológica, Universidade Federal de Santa Maria, Santa Maria, Rio Grande do Sul, Brasil; 3 Laboratório de Neurociências, Instituto de Pesquisas Biomédicas e Instituto do Cérebro, Pontifícia Universidade Católica do Rio Grande do Sul, Porto Alegre, Rio Grande do Sul, Brasil; 4 Faculdade de Biociências, Departamento de Ciências Fisiológica, Pontifícia Universidade Católica do Rio Grande do Sul, Porto Alegre, Rio Grande do Sul, Brasil; 5 Centro de Educação Física e Desportos, Departamento de Métodos e Técnicas Desportivas, Laboratório de Bioquímica do Exercício (BIOEX), Universidade Federal de Santa Maria, Santa Maria, Rio Grande do Sul, Brasil; 6 Centro de Ciências da Saúde Programa de Pós-Graduação em Farmacologia, Departamento de Fisiologia and Farmacologia, Universidade Federal de Santa Maria, Santa Maria, Rio Grande do Sul, Brasil; 7 Universidade Regional Integrada do Alto Uruguai e das Missões, URI - Campus Santiago, Santiago, Rio Grande do Sul, Brasil; Universidade Federal do Rio de Janeiro, Brazil

## Abstract

**Background and Aims:**

Glutaric aciduria type I (GA-I) is characterized by accumulation of glutaric acid (GA) and neurological symptoms, such as cognitive impairment. Although this disease is related to oxidative stress and inflammation, it is not known whether these processes facilitate the memory impairment. Our objective was to investigate the performance of rat pups chronically injected with GA and lipopolysaccharide (LPS) in spatial memory test, antioxidant defenses, cytokines levels, Na+, K+-ATPase activity, and hippocampal volume. We also evaluated the effect of N-acetylcysteine (NAC) on theses markers.

**Methods:**

Rat pups were injected with GA (5umol g of body weight-1, subcutaneously; twice per day; from 5th to 28th day of life), and were supplemented with NAC (150mg/kg/day; intragastric gavage; for the same period). LPS (2mg/kg; *E.coli* 055 B5) or vehicle (saline 0.9%) was injected intraperitoneally, once per day, from 25th to 28th day of life. Oxidative stress and inflammatory biomarkers as well as hippocampal volume were assessed.

**Results:**

GA caused spatial learning deficit in the Barnes maze and LPS potentiated this effect. GA and LPS increased TNF-α and IL-1β levels. The co-administration of these compounds potentiated the increase of IL-1β levels but not TNF-α levels in the hippocampus. GA and LPS increased TBARS (thiobarbituric acid-reactive substance) content, reduced antioxidant defenses and inhibited Na+, K+-ATPase activity. GA and LPS co-administration did not have additive effect on oxidative stress markers and Na+, K+ pump. The hippocampal volume did not change after GA or LPS administration. NAC protected against impairment of spatial learning and increase of cytokines levels. NAC Also protected against inhibition of Na+,K+-ATPase activity and oxidative markers.

**Conclusions:**

These results suggest that inflammatory and oxidative markers may underlie at least in part of the neuropathology of GA-I in this model. Thus, NAC could represent a possible adjuvant therapy in treatment of children with GA-I.

## Introduction

GA-I is an inherited neurometabolic disease caused by a deficiency of mitochondrial enzyme glutaryl-CoA dehydrogenase (GCDH; EC 1.3.99.7) involved in the metabolism of L-lysine, L-hydroxylysine and L-tryptophan. Affected patients usually present a major accumulation of GA and a lesser amount of 3-hydroxyglutaric acid (3-OH-GA) and glutaconic acid in the body fluids [1-4]. The symptoms and signs of GA-I are predominantly neurological and include seizures, dystonia, dyskinesia, memory impairment, and irreversible cerebral injury, especially during crises of metabolic decompensation [5-7]. It is feasible that during these episodes, which usually follow infections or other metabolic stress conditions, the increased tissue levels of GA due to highly accelerated endogenous proteolysis may trigger molecular mechanisms leading to neurological dysfunction. In fact, affected infants may become debilitated and septic rather quickly [8], and sepsis may contribute to their neurologic deterioration.

Although the neurological disorders are prevalent in GA-I, little is known about the mechanism by which the accumulated organic acids lead to these alterations after infection process [9-11]. Memory impairment, studies have reported that patients with GA-I present cognitive deficit [5-7] mainly after encephalopathic crises precipitated by a non-specific illness or infection [10], which suggests a close association between inflammation and the occurrence of cognitive disorder in GA-I children. 

In this context, studies have reported a correlation between elevated levels of proinflammatory cytokines and cognitive deficits after neuroinflammatory processes [12,13]. Moreover, a large number of cognitive disorders in humans and animal models are associated with elevated levels of proinflammatory molecules, such as IL-1β and TNF-α [12-19].

Besides neuroinflammatory processes, oxidative stress has been also implicated as an important causative factor in various neurodegenerative and neurometabolic diseases, including glutaric acidemia [9,11,20-22]. Although the mechanisms underlying brain damage in GA-I are not well established, growing evidence suggests that excitotoxicity [23,24], Na^+^,K^+^-ATPase activity inhibition [9,20], inflammation [11], and free radical generation [9,22] play a central role in the neuropathogenesis of the GA-I.

In this context drugs with antioxidant and antinflammatory potential could be consequently a promoting approach to neuroprotection in patients with glutaric acidemia [9,11,20,22]. This seems to be the case of NAC, an agent with antioxidant and antinflammatory properties, which inhibits oxidative metabolism and decreases the release of proinflammatory molecules [25-27]. Studies have also shown that NAC prevents memory impairment in some experimental models [28].

Since there is clinical evidence that infection and/or inflammation mediators facilitate metabolic crises and neurologic dysfunction (including cognitive impairment) in patients with GA-I, in this study we investigated the performance of rat pups chronically injected with GA and LPS in spatial memory test, antioxidant defenses, cytokines levels, NA^+^,K^+^-ATPase activity, and hippocampal volume. Furthermore, we also evaluated whether NAC could improve these behavioral, biochemical or structural changes induced by GA and LPS administration.

## Materials and Methods

### Ethics Statement

Laboratory experiments were performed in accordance with national and international legislations (Brazilian College of Animal Experimentation [COBEA] and the U.S. Public Health Service’s Policy on Humane Care and Use of Laboratory Animals-PHS Policy) and approved by the Ethics Committee for Animal Research of Universidade Federal de Santa Maria (UFSM; Permit Number: 116/2010). Indeed, animal handling and laboratory assays were carried out in such a way that all efforts were made to minimize suffering.

### Reagents

Unless otherwise stated, reagents were purchased from Sigma (St. Louis, MO, USA)

### Animals

The present study utilized Wistar rats with 5 days of life. Pregnant Wistar rats were housed in individual cages and left undisturbed during gestation. Forty-eight hours after delivery, litters were culled to eight male pups. Pups were fed by the mother since birth until 21 days of life when they were weaned. Animals were divided in order to have the same number of rats for each treatment in each cage. Animals had free access to water and to a standard commercial chow and were maintained on a 12:12 h light/dark cycle in an air-conditioned constant temperature (24 ± 1°C, 55% relative humidity) colony room. The “Principles of Laboratory Animal Care” (NIH publication no. 80-23, revised 1996) were followed in all experiments and the experimental protocol was approved by the Ethics Committee for Animal Research of the Federal University of Santa Maria, Santa Maria, Brazil. All efforts were made to minimize the number of animals used and their suffering.

### Drugs and injections

#### GA injection

Buffered GA, pH 7.4 (5 µmol g of body weight^−1^) was administered subcutaneously, twice a day, from 5^th^ to 28^th^ day of life to produce brain concentrations of GA around 0.6 µmol g^−1^, ~0.72 mM [29] similar to concentrations found in patients with GA-I. Control animals received saline subcutaneously in the same volumes and frequency. All solutions were prepared so that each animal received 10 µL solution g of body weight ^−1^.

#### LPS injection

As GA-I patients may become debilitated after an infection process and since sepsis may contribute to their neurologic deterioration [8], the rat pups were injected intraperitoneally (i.p.) with LPS (2 mg/kg; *E.coli* 055 B5) [30,31] or vehicle (saline 0.9%), once per day, on postnatal days 25-28 to mimic a severe infection state [32].

### NAC administration

Rat pups were supplemented with NAC (150mg/kg/day) or vehicle (saline 0.9%) by intragastric gavage once a day on postnatal days 5-28 [33].

### Physical development

All rats used in experiments had their behavioral development assessed. For this, the weight of animals was daily determined at the appropriate ages by one experimenter who was not aware of the subject condition.

### Cognitive tasks

Behavioral experiments were carried out between 9:00 am and 2:00 pm. Animals were assessed on the Barnes maze on postnatal days 29-32. After Barnes maze test, animals were tested in the open ﬁeld task on postnatal day 32.

### Barnes Maze task

The next day after the end of treatment animals were trained to solve the Barnes maze. The Barnes maze is a validated test often used for the assessment of spatial learning and memory in rodents [34].The Barnes maze paradigm exploits the natural inclination of small rodents to seek escape to a darkly lit sheltered environment when placed in an open arena under bright and aversive illumination. Our maze consists of a 120 cm diameter circular wooden table, 3.5 cm thick and elevated 90 cm above the floor.

Twenty holes (6 cm diameter) were equidistantly located around the perimeter and centered 5 cm from it. The apparatus was located in a 4 m x 4 m test room where four visuospatial cues made of rigid black paper (rectangle, circle, cross, and triangle) were affixed to the walls but not directly over any maze hole; this increases the spatial component of the Barnes maze during training [35]. A black wooden escape tunnel (15 cm x 10 cm x 30 cm) was placed beneath one randomly selected hole for each rat but remained constant throughout the training sessions for a given rat. The remaining 19 holes led only to a false escape box (15cm x 10cm x 10cm), which from the platform, appeared indistinguishable from an escape box but was too small to be entered. False boxes removed visual cues that might be observed through an open hole. There was a bright illumination of 300 lux over the maze.

On the first day of the experiment the rats were moved to a testing room and left undisturbed for 60 min. Following this habituation period the rats were trained to find the escape hole. They were placed in the escape box for 1 min and then into a cylindrical opaque chamber (start box) in the center of the maze. With light on, the start box was removed and the rats were allowed to explore freely and find the escape box. The maximum time allowed to find the escape was 180 s. Each rat was given three trials per day over four consecutive days. In each trial we recorded the time to reach the escape tunnel and the number of wrong holes visited. A visited hole was considered when the animal poked at a hole. The arena and the boxes were wiped clean using distillated water both between each training session for a given rat and between each rat. Immediately after the second training session on the Barnes maze, animals were transferred to an open-field measuring 50 x 60 cm, with the floor divided into 12 squares measuring 12 x 12 cm each. The open-field test was carried out to identify motor disabilities and lasted for 5 min. During this time an observer who was not aware of pharmacological treatments recorded the number of crossing and rearing responses manually.

### Open-ﬁeld task

After the fourth day of Barnes maze test the locomotor activity was measured for 5 min in the open field. The apparatus consisted of a wooden box measuring 60 cm x 40 cm x 50 cm with a glass front wall. Its ﬂoor was divided by black lines into 12 equal squares. Animals were gently placed facing the rear left corner of the arena and the number of squares crossed with the four paws recorded for 5 min to evaluate motor activity [36]. The testing room was dimly illuminated with indirect white lighting.

### Elevated plus maze task

Based on the design of File and Gonzalez [37] the maze consisted of two opposite closed arms (30 cm x 5 cm) enclosed with walls (15 cm in height) and two opposite open arms (also 30 cm x 5 cm, without edges) forming a plus shape. The whole apparatus had a central arena (5 cm x 5 cm) and was elevated to 80 cm above the floor by a tripod. Each rat was placed in the central arena of the maze facing an open arm and observed for 5 min. The behaviors recorded were: total number of entries, the percentage of time spent on either arm, and percentage of time spent in the middle. The apparatus was cleaned thoroughly between the 5 min observation sessions with a 30 % ethanol solution.

### Sample processing

After the behavioral evaluation (second day of Barnes Maze test), a subset of animals was killed by decapitation, and hippocampi were rapidly removed, dissected and homogenized in 1-ml sterile PBS, and then stored at -70 °C until processed further.

### IL-1β and TNF-α immunoassay

To analyze the content of IL-1β and TNF-α the hippocampus was weighted and homogenized (1:10) in a solution containing bovine serum albumin (BSA 10 mg/ml), EGTA (2 mM), EDTA (2 mM) and PMSF (0.2 mM) in phosphate-buffered saline (PBS, pH 7.4) using a Potter homogenizer. The hippocampus homogenate was centrifuged (3000 g for 10 min) and cytokines were determined in supernatant. Cytokine levels were measured using a commercially available ELISA Kit from Rand D Systems (Minneapolis, MN) using an antibody selective against rat IL-1β or TNF-α, according to the manufacturer’s protocol. Results are expressed in pg/mg of protein for hippocampus homogenate assays. Absorbance was read at 405 nm. The detection limit was 4 ng/ml.

### Measurement of TBARS content

Thiobarbituric Acid Reactive Substances (TBARS) content was estimated in a medium containing 0.2 ml of brain homogenate, 0.1 ml of 8.1% SDS, 0.4 ml of acetic acid buffer (500 mM, pH 3.4), and 0.75 ml of 0.81% (TBA). The mixture was finally made up to 2 ml with type I ultrapure water and heated at 95 °C for 90 min in a water bath using a glass ball as a condenser. After cooling to room temperature, absorbance was measured in the supernatant at 532 nm [38].

### Non Protein Thiol determination

Non Protein Thiols (NPSH) were measured spectrophotometrically with Ellman’s reagent [39]. An aliquot of 200 μL for hippocampus in a final volume of 900 μL of solution was used for the reaction. The reaction product was measured at 412 nm after the addition of 10 mM 5-5-dithio-bis (2-nitrobenzoic acid) (DTNB) (0.05 mL). A standard curve using cysteine was added to calculate the content of thiol groups in the samples, and was expressed as µmol SH/g tissue. 

### Catalase activity determination

For the catalase (CAT) assay in hippocampus, the tissue was homogenized in 50 mM potassium phosphate buffer, pH 7.5, at a proportion of 1:9 (w/v) and 1:5 (w/v), respectively. The homogenate was centrifuged at 2000 g for 10 min to yield a supernatant that was used for the enzyme assay [40]. The reaction mixture contained 50 mM potassium phosphate buffer (pH 7), 10 mM H_2_O_2_, and 20 μL of the supernatant. The rate of H_2_O_2_ reaction was monitored at 240 nm for 2 min at room temperature as determined in total blood assay. The enzymatic activity was expressed in units mg^−1^ protein (One unit of the enzyme is considered the amount of CAT which decomposes 1 μmol of H_2_O_2_ per min at pH 7 at 25 °C.)

### Superoxide dismutase activity determination

With the purpose of performing the superoxide dismutase (SOD) assay in hippocampus, the tissue was adequately diluted with Tris-HCl pH 7.4 at a proportion of 1:40 (w/v) and 1:60(w/v) respectively [41]. Briefly, epinephrine undergoes auto-oxidation at pH 10.2 to produce adrenochrome, a colored product that was detected at 480 nm. The addition of samples (10, 20, 30 µL) containing SOD inhibits the auto-oxidation of epinephrine. The rate of inhibition was monitored for 180 s. The amount of enzyme required to produce 50% inhibition was defined as one unit of enzyme activity. 

### Glutathione levels

The levels of glutathione (GSH) were determined fluorometrically as described by Hissin and Hilf [42] with minor modifications using 0-phthaladehyde (OPA) as fluorophore. Briefly, the hippocampus was homogenized in 0.1M HClO_4_. Homogenates were centrifuged at 2500 g for 10 min and the low-speed supernatants were separated for measurement of GSH. The supernatant (100 μl) was incubated with 100 ìl of OPA (0.1% in methanol) and 1.8 ml of 0.1M phosphate buffer (pH 8.0) for 15 min at room temperature in the dark. Fluorescence was measured with a fluorescence spectrophotometer at excitation wavelength of 350 nm and at emission wavelength of 420 nm. GSH levels were expressed as nmol GSH/g of tissue.

### Na^+^, K^+^-ATPase activity measurements

 The Na^+^,K^+^-ATPase activity was measured according to Wyse et al.[43]. Brieﬂy, the assay medium consisted of 30 mM Tris-HCl buffer, pH 7.4; 0.1 mM EDTA, 50 mMNaCl, 5 mMKCl, 6 mM MgCl_2_, and 50 µg of protein in the presence or absence of ouabain (1 mM) in a ﬁnal volume of 350 µL. The reaction was started by the addition of adenosine triphosphate to a ﬁnal concentration of 5 mM. After 30 min at 37 °C, the reaction was stopped by the addition of 70 µL of 50% (w/v) trichloroacetic acid. Saturating substrate concentrations were used, and the reaction was linear with protein and time. Appropriate controls were included in the assays for non-enzymatic hydrolysis of ATP. The amount of inorganic phosphate (Pi) released was quantiﬁed by the colorimetric method described by Fiske and Subbarow [44] using KH_2_PO_4_ as reference standard. Speciﬁc Na^+^,K^+^-ATPase activity was calculated by subtracting the ouabain-insensitive activity from the overall activity (in the absence of ouabain) and expressed in nmol Pi/mg protein/min.

 In a separate set of experiments we investigated whether some Na^+^,K^+^-ATPase isoform is selectively inhibited. For this purpose we used a classical pharmacological approach based on the isoform-speciﬁc sensitivity to ouabain [45]. We determined whether some treatments inhibited ouabain-sensitive ATPase activity using 3 µM (that inhibits Na^+^,K^+^-ATPase isoforms containing α2 and α3 subunits) or 4 mM ouabain (that inhibits all isoforms).

### Hippocampal Volume

Considering that hippocampus cannot act as a unitary structure and that dorsal portion is related to learning processes and memory [93], we decided to investigate the effect of GA, LPS and NAC treatment on the volume of dorsal hippocampus. In our study the dorsal hippocampus was defined as the hippocampus comprised between the following Paxinos and Watson atlas coordinates: Bregma -1.60mm/Interaural 7.40mm; Bregma -3.8mm/Interaural 5.2mm. We analyzed this hippocampal region because it is deeply associated to learning and memory processes and presents well defined anatomical limits, being suitable to unbiased volume estimation using Cavalieri principle. For this propose, after the behavioral evaluation (second day of Barnes Maze test), a subset of animals was deeply anesthetized with thiopental (40 mg/kg, i.p.) and transcardially perfused with saline solution followed by a solution of 4% paraformaldehyde in 0.1 M phosphate buffer, pH 7.4. After perfusion, the brains were removed from the skulls, post-fixed in the same solution at room temperature for 24 h and cryoprotected by immersion in 30% sucrose solution in phosphate buffer at 4 °C until they sank. After these procedures, the brains were quickly frozen in isopentane, previously cooled in liquid nitrogen (-70°C). For each brain, coronal sections from the hippocampus (60µm) were obtained using a cryostat (Leica CM 1850) at -20° C and mounted on gelatinized slides. These sections were identified according to Paxinos and Watson’s Atlas (coordinates: interaural 7.2 mm, bregma -1.8 mm and interaural 5.4 mm, bregma -3.6 mm). Slides were hydrated in decreasing ethanol solutions (absolute, 80%, 70%) and distilled water. They were placed in acresyl violet solution acetified 0.01% for approximately 1 min. Then, they were dehydrated in increasing alcohol solutions (70%, 80% absolute) and clearedin xylene, and finally covered with coverslips with entellan resin.

### Volume estimation of dorsal hippocampus

Sections of dorsal hippocampus were digitized using an Olympus BX50 microscope coupled to a Motic Images Plus 2.0 camera and Image Pro Plus Software 6.1 (Media Cybernetics, CA, USA). The unbiased volume estimation of the dorsal hippocampus was performed using the Cavalieri principle associated to the counting point method. The volume was estimated using the following equation:V=T·a/p·ΣP, where, V=Volume estimation; T=Distance between the analyzed sections (240μm); a/p=point area (1mm^2^)and ΣP is the sum of points overlaid in the image [46-48]. Two histology specialists blinded to the source of the images carried out the volume estimations.

### Protein determination

Protein content was measured colorimetrically by the method of Bradford [49] using bovine serum albumin (1 mg/ml) as a standard.

### Statistical Analysis

The statistical analysis was carried out by two or three-way analysis of variance (ANOVA) and only F values of P <0.05 are presented. Post hoc analysis was carried out by Duncan’s multiple comparisons test, when appropriate. All data were expressed as mean ± SEM. Statistical analyses were performed utilizing the SPSS software in a PC-compatible computer.

## Results

### Physical Development of Animals

**Figure 1:**
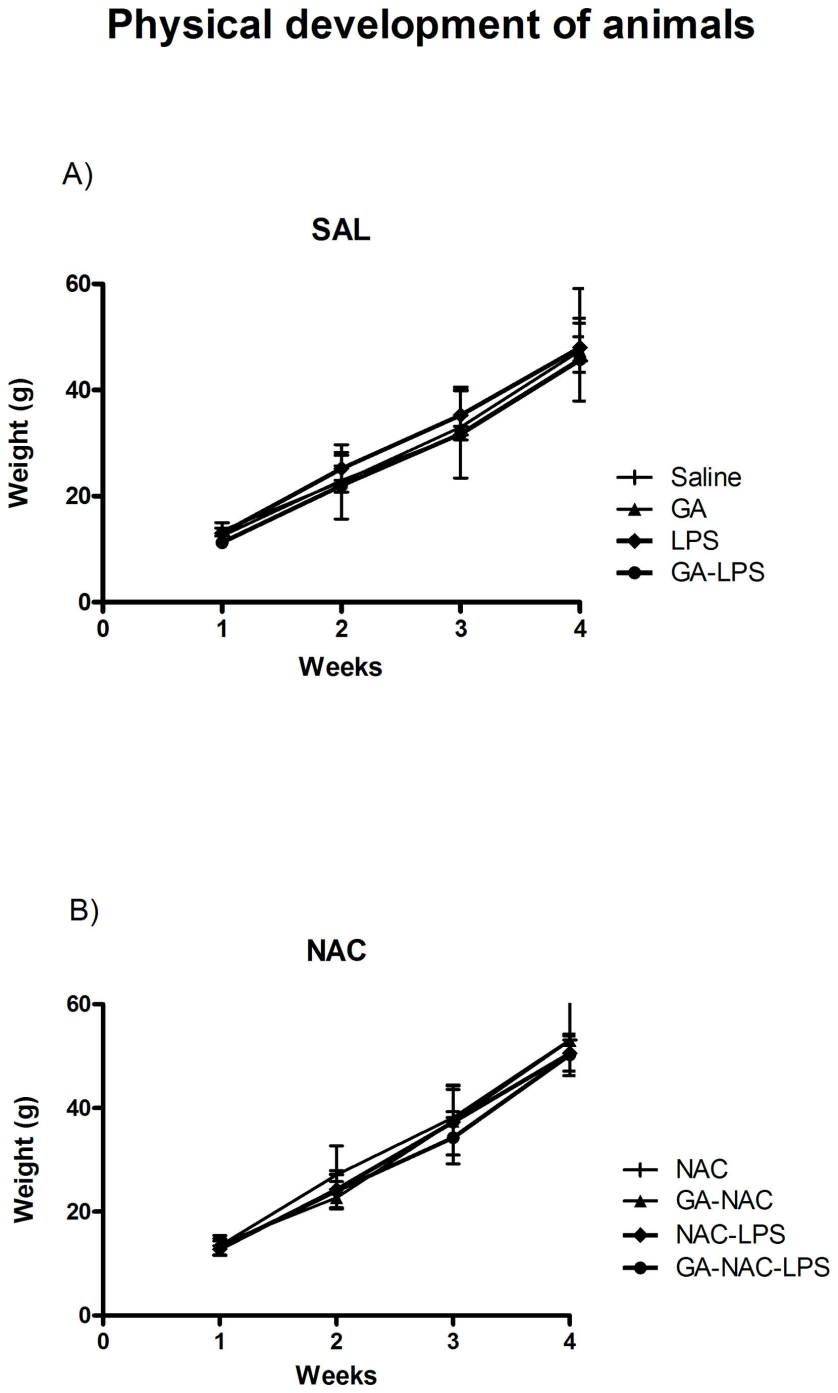
Effect of early postnatal chronic GA, NAC and LPS administration on physical development of animals. Data are presented as means ± S.E.M. for n = 8–9 in each group. No significant differences between groups were detected.

The physical development of animals determined by their weights during the treatment is shown in Figure 1A and 1B. Statistical analysis of the weight of animals (three-way ANOVA) did not show a significant drug (Saline or GA) by toxin (Saline or LPS) by treatment (Sal or NAC) interaction: [F(1,57)= 0.46; p> 0.05].

### Effect of NAC on Open field task

**Figure 2:**
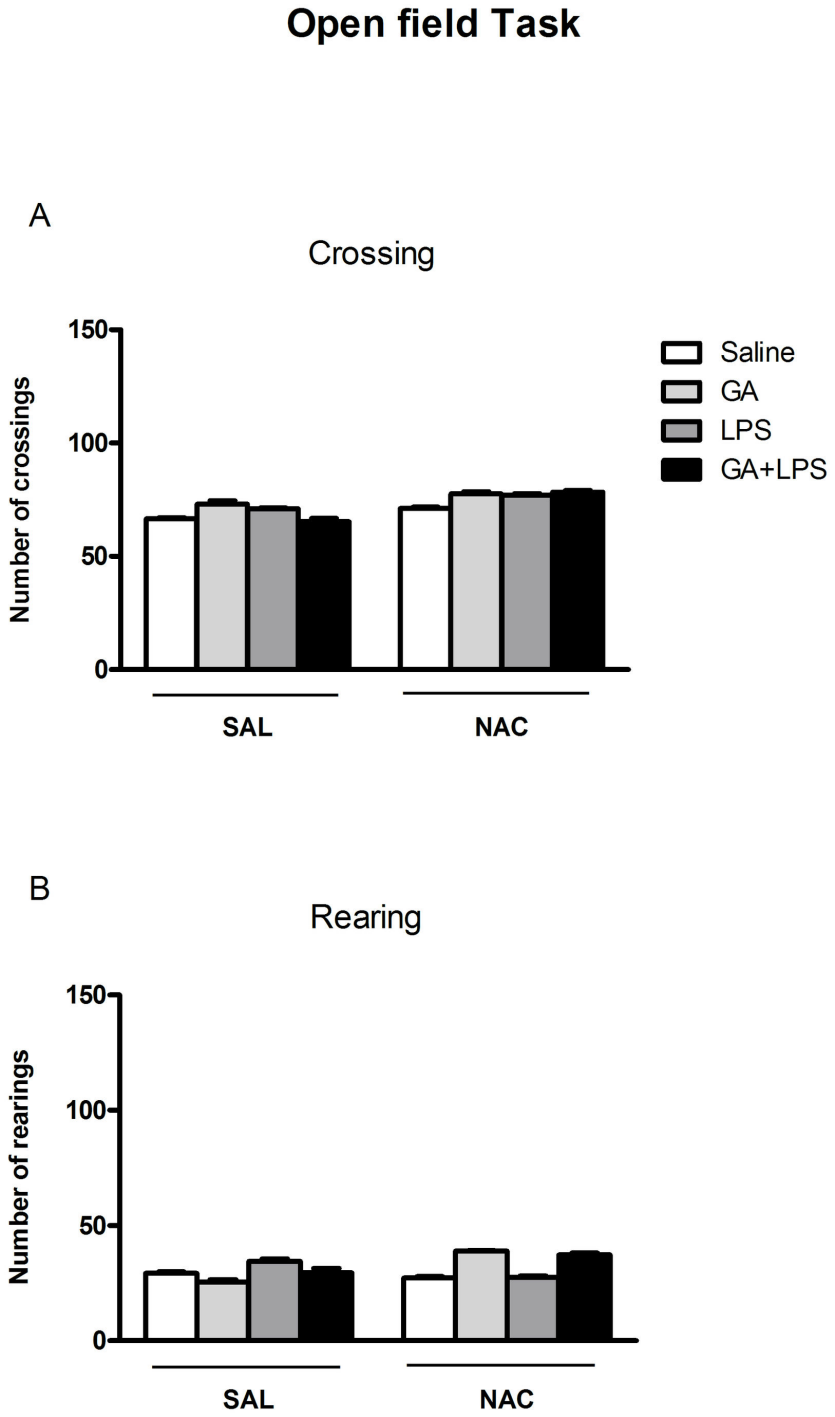
Effect of early postnatal chronic GA, NAC and LPS administration on the number of crossing (A) and rearing (B) of the animals. Data are presented as means ± S.E.M. for n = 8-9 in each group. No significant differences between groups were detected.

 The exploratory and locomotor activity of animals is shown in Figure 2. Statistical analysis of the number of crossing and rearing (three-way ANOVA) did not show a significant drug (Saline or GA) by toxin (Saline or LPS) by treatment (Sal or NAC) interaction: [F(1,57)= 1.00; p>0.05; Figure 2A and F(1,57)= 1.00; p >0.05; Figure 2B; respectively).

### Effect of NAC on Spatial Memory

**Figure 3:**
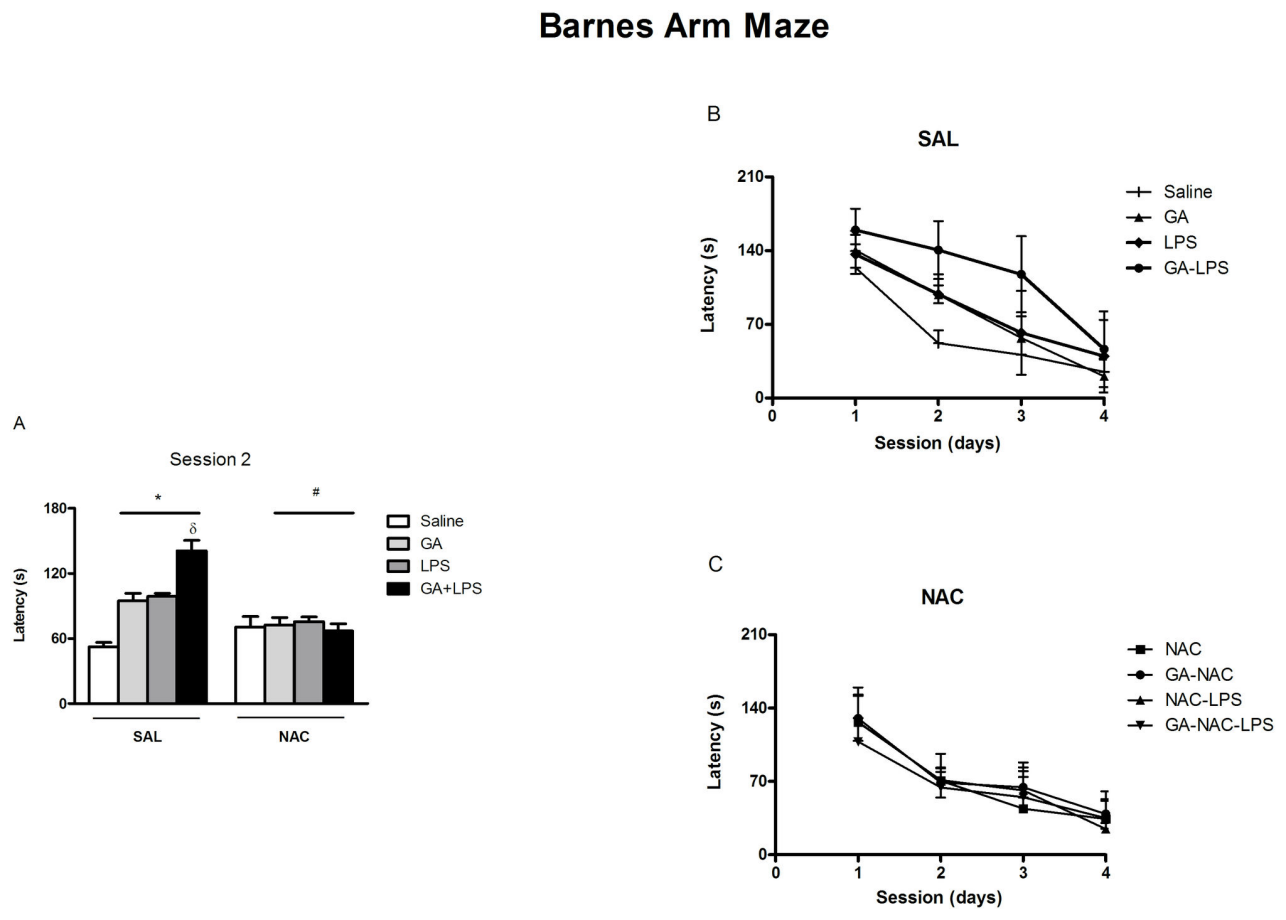
NAC prevents the memory deficit induced by GA and LPS measured as escape latency of rat pups in the Barnes maze. *P < 0.05 compared with saline treated group, ^#^P< 0.05 compared with respective control group and ^δ^P < 0.001 compared with GA-treated group (Duncan’s multiple comparisons test). Data are presented as means ± S.E.M. for n = 7-9 in each group. Figures 3B and 3C show an amplification of the second day of test.

The effect of GA and LPS treatment on cognitive performance is shown in Figure 3B. Statistical analysis demonstrated that all the animals [*F*(1,57)=14.33; p <0.001] decreased the latency to escape indicating that they have learned the task. 

In addition, statistical analysis of the latency to escape revealed that GA and LPS co-administration potentiated the memory impairment in the Barnes task [*F*(1,57)=27.85; p <0.001]. On the other hand, statistical analysis (three-way analysis of variance (ANOVA) revealed a significant drug (Saline or GA) by toxin (Saline or LPS) by treatment (Sal or NAC) interaction on the second [F(1,57)=5.28; p <0.05] and third days of test F(1,57)= 3.95; p<0.05; Figure 3 A], indicating a decreased performance of these animals in this spatial learning paradigm. These results revealed that NAC administration decreased the latency to escape induced by GA and LPS co-administration. Considering that GA, LPS and NAC presented effects mainly on the second day of spatial memory testing, the next experiments carried out were limited to 2 days.

### Effect of NAC on anxiety

Statistical analysis of the percent of time and entries in open arm in the elevated plus mazeb (three-way ANOVA) did not show a significant drug (Saline or GA) by toxin (Saline or LPS) by treatment (Sal or NAC) interaction: [F(1,57) = 0.43; p>0.05 and [F(1,57) = 0.27; p >0.05; respectively]. In addition, statistical analysis (three-way ANOVA) did not show a significant drug (Saline or GA) by toxin (Saline or LPS) by treatment (Sal or NAC) interaction for percent of time [F(1,57) = 0.85; p> 0.05], number of entries into the enclosed arm [F(1,57) = 0.34; p > 0.05] and time spent on middle [F(1,57) = 0.34; p >0.05], indicating that the treatment had no effect on anxiety-like behavior (Table 1). 

**Table 1:** Effect of early postnatal chronic GA, NAC and LPS administration on anxiolytic-like behavior of rat pups on the second day of Barnes.

Group	Sal	GA	LPS	GA-LPS	Sal	GA	LPS	GA-LPS
		SAL				NAC		
%T.O	8.5 + 1.9	6.11 + 2.5	14.4 + 2.3	7.3 + 3.7	7.07 + 2.3	14.2 + 4.2	13.9 + 4.2	17.5 + 6.0
%No.E.O	30.8 + 4.7	26.6 + 2.4	25.0 + 5.9	28.3 + 6.7	30.1 + 4.3	35.2 + 8.0	29.8 + 7.9	31.0 + 5.9
%T.E	82.2 + 3.0	84.6+ 4.6	76.0 + 3.8	82.0 + 5.0	80.9 + 1.76	63.9 + 9.0	76.5 + 5.2	67.7 + 6.0
%No.E.E	69.0 + 4.7	73.2 + 2.4	74.8 + 5.9	71.6 + 6.7	69.8 + 4.3	64.7 + 8.0	70.1 + 7.9	68.9 + 5.9
%T.M	8.9 + 1.5	9.1 + 2.7	10.7 + 2.0	10.4 + 2.4	7.7 + 1.2	21.6 + 6.4	9.4 + 2.9	13.9 + 2.3
N	8	7	7	7	7	7	7	7

Data are presented as means ± S.E.M. for n = 7-8 in each group. No significant differences between groups were detected. %T.O percent of time spent on open arms; %No.E.o percent of number of entries on open arms; %T.E percent of time spent on closed arms; % No.E.R percent of number of entries on encloses arms; %T.M percent of time spent on the middle.

### Effect of NAC on IL-1β and TNF-α levels

Since numerous studies have found a link between elevated levels of cytokines and memory deficits after neuroinflammatory processes [12,13], we decided to determine the levels of IL-1β and TNF-α in hippocampus of rat pups. 

**Figure 4:**
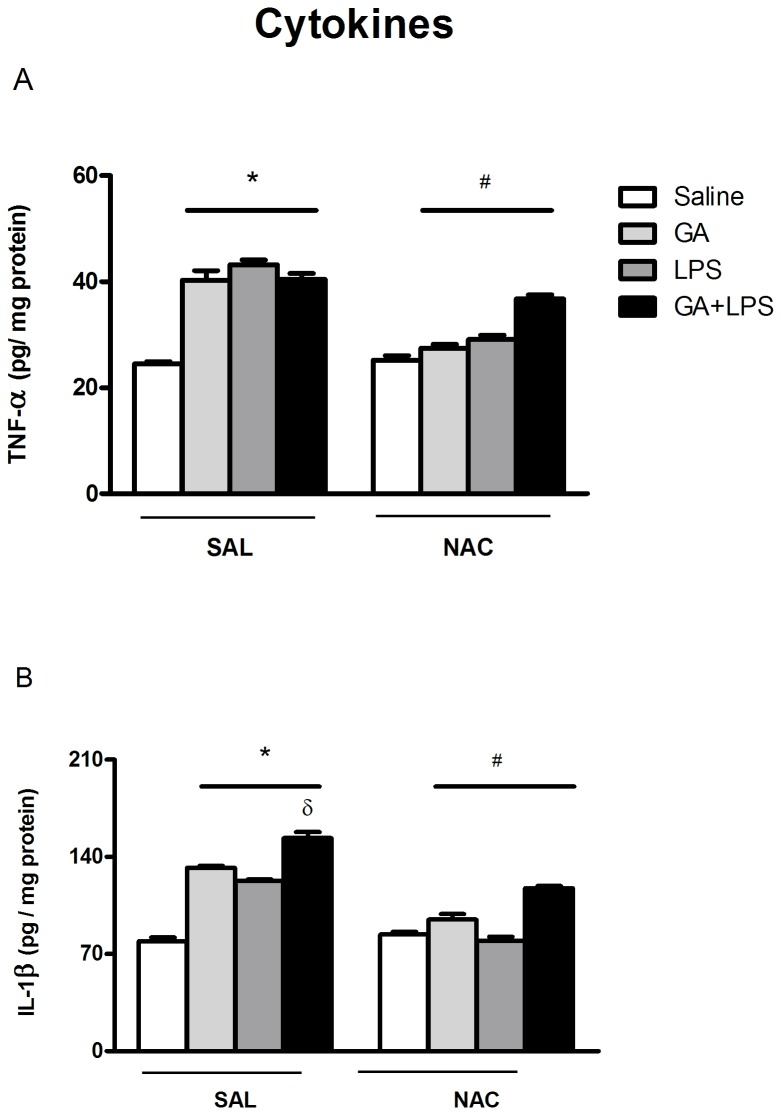
Effect of early postnatal chronic GA, NAC and LPS administration on cytokine levels on the second day of Barnes maze. NAC prevented the increase in TNF-α (A) and IL-1β (B) levels. *P < 0.001 compared with saline treated group, ^#^P< 0.001 compared with the respective control group and ^δ^P < 0.05 compared with GA-treated group (Duncan’s multiple comparisons test). Data are presented as means ± S.E.M. for n = 6-7 in each group.

TNF-α levels in the hippocampus are shown in Figure 4A. The statistical analysis (two-way ANOVA) revealed an increase in TNF-α levels induced by GA [*F*(1,44)=22.83; p <0.001] and LPS [*F*(1,44)=14.93; p <0.001] when compared with de control group. Statistical analysis (two-way ANOVA) also revealed an increase in IL-1β levels induced by GA [*F*(1,42)=38.86; p <0.001] and LPS [*F*(1,42)=26.15; p <0.001] when compared with the control group (Figure 4B). The GA and LPS co-administration had no additive effects in TNF-α [*F*(1,44)=0.13; p >0.05] levels. However, this treatment potentiated the increase of IL-1β levels [*F*(1,42)=0.01; p >0.05] in the hippocampus of rat pups.

Furthermore, Figure 4 (A and B) shows NAC effect on TNF-α and IL-1β levels in hippocampus of rat pups. Statistical analysis (three-way ANOVA) revealed that NAC administration reduced TNF-α levels [significant drug (Saline or GA) by toxin (Saline or LPS) by treatment (Sal or NAC) interaction: *F*(1,44)=3.7; p <0.05] and IL-1β levels increase [significant drug (Saline or GA) by toxin (Saline or LPS) by treatment (Sal or NAC) interaction: *F*(1,44)=5.73; p <0.05; respectively] induced by GA and LPS in the hippocampus of rat pups.

### Effect of NAC on Antioxidant Defenses

#### TBARS content

**Figure 5:**
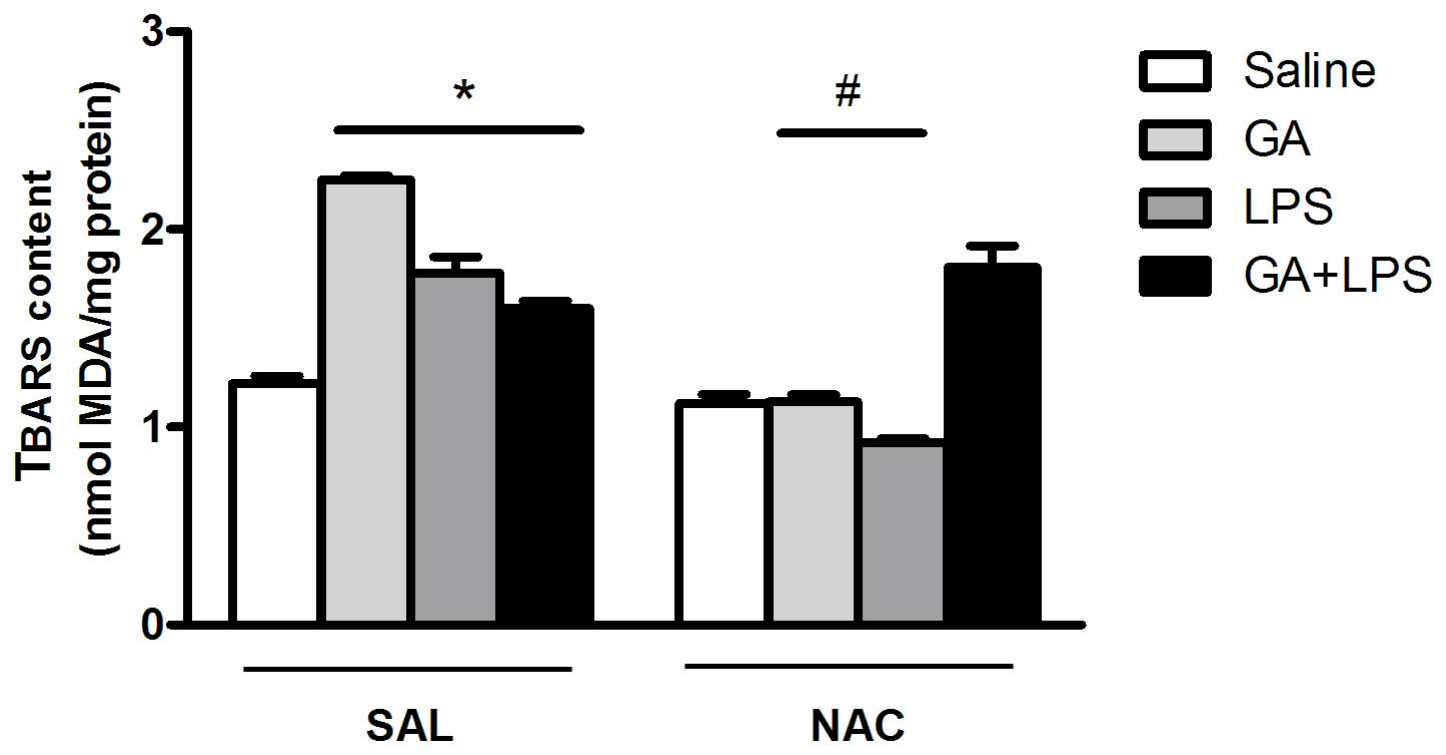
Effect of early postnatal chronic GA, NAC and LPS administration on TBARS content. NAC prevented the increase of TBARS content induced by GA and LPS. *P < 0.01 compared with saline treated group and ^#^P< 0.05 compared with respective control group (Duncan’s multiple comparisons test). Data are presented as means ± S.E.M. for n = 7 in each group.

Statistical analysis on TBARS content (three-way ANOVA) showed a significant drug (Saline or GA) by toxin (Saline or LPS) by treatment (Sal or NAC) interaction: [F(1,48)=4.32; p <0.05] (Figure 5). Post hoc analysis showed that the GA and LPS administration increased TBARS content and that systemic NAC administration prevented the GA and LPS-induced TBARS content increase.

#### NPSH content

**Figure 6:**
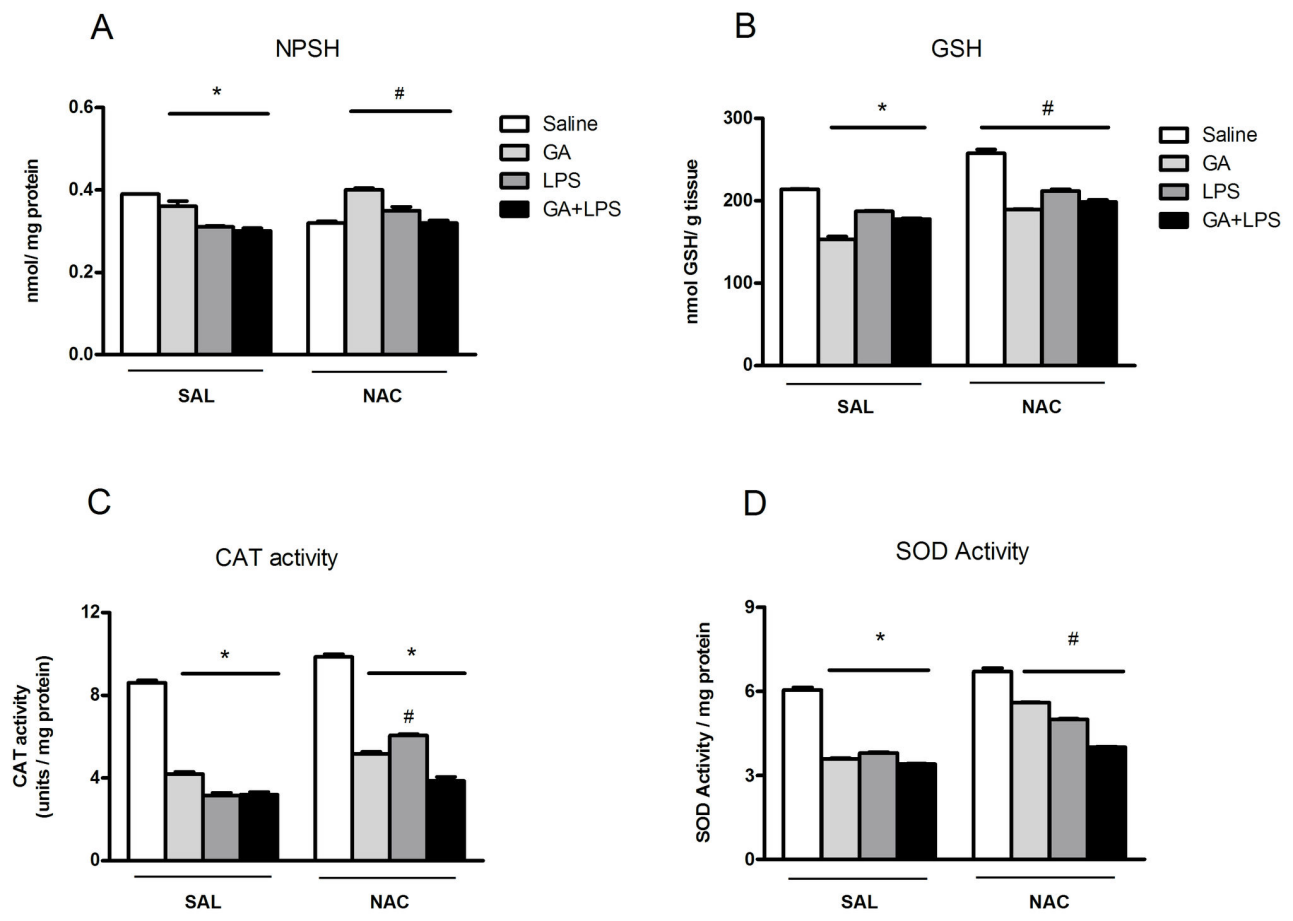
Effect of early postnatal chronic GA, NAC and LPS administration on NPSH content, GSH level, CAT and SOD activities. NAC prevented the decrease of antioxidant defenses induced by GA and LPS.*P < 0.001 compared with saline treated group and ^#^P< 0.001 compared with respective control group (Duncan’s multiple comparisons test). Data are presented as means ± S.E.M. for n = 7 in each group.

Statistical analysis on SH content (three-way ANOVA) showed a significant drug (Saline or GA) by toxin (Saline or LPS) by treatment (Sal or NAC) interaction: [*F*(1,44)=6.9; p <0.05] (Figure 6A). Post hoc analysis showed that the GA and LPS administration decreased SH content and that systemic NAC administration prevented this effect.

#### Glutathione levels

Statistical analysis on GSH levels (three-way ANOVA) showed a significant drug (Saline or GA) by toxin (Saline or LPS) by treatment (Sal or NAC) interaction: [*F*(1,48)=32.62; p <0.001] (Figure 6C). Post hoc analysis showed that the GA and LPS administration decreased GSH levels and that systemic NAC administration prevented this effect (Figure 6B).

#### CAT activity

Statistical analysis on the CAT activity (three-way ANOVA) showed a significant drug (Saline or GA) by toxin (Saline or LPS) by treatment (Sal or NAC) interaction: [*F*(1,48)=14.5; p <0.001] (Figure 6C). Post hoc analysis showed that the GA and LPS administration decreased CAT activity and that systemic NAC administration prevented this effect. 

#### SOD activity

Statistical analysis on the SOD activity (three-way ANOVA) showed a significant drug (Saline or GA) by toxin (Saline or LPS) by treatment (Sal or NAC) interaction: [*F*(1,25)=11,90; p <0.001] (Figure 6D). Post hoc analysis showed that the GA and LPS administration decreased SOD activity and that systemic NAC administration prevented this effect.

### Effect of NAC on Na^+^, K^+^-ATPase activity

**Figure 7:**
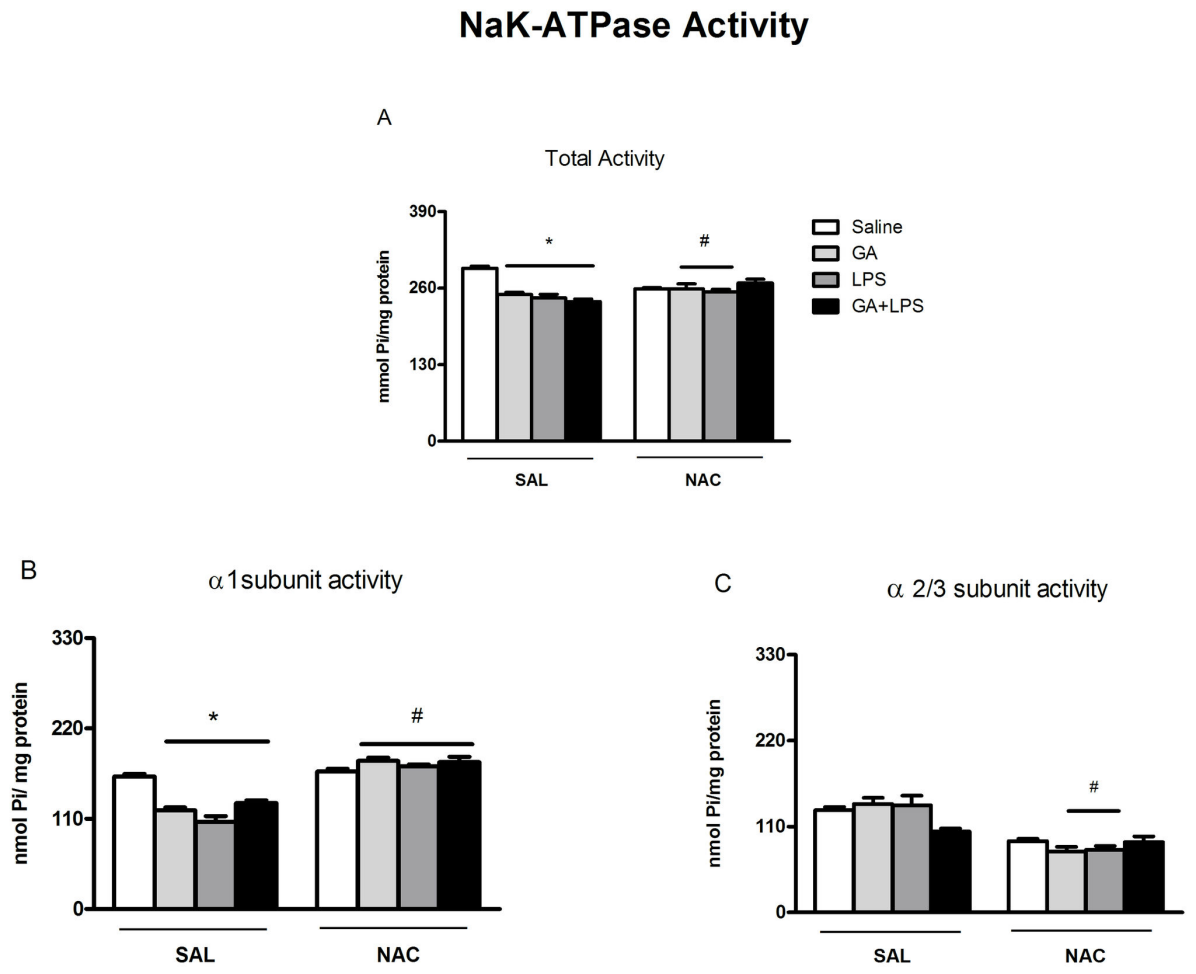
NAC prevented the decrease of α1 subunit activity of Na^+^,K^+^-ATPase enzyme. Effect of early postnatal chronic GA, NAC and LPS administration on Na^+^,K^+^-ATPase total activity (A); on α1 subunit activity of Na^+^,K^+^-ATPase enzyme (B); and on α2/3 subunit activity of Na^+^,K^+^-ATPase enzyme (C), on the second day of Barnes maze.*P < 0.001 compared with saline traded group and ^#^P< 0.001 compared with respective control group (Student–Newman–Keuls test). Data are presented as means ± S.E.M. for n = 7-9 in each group.

Statistical analysis (two-way ANOVA) showed that GA [*F*(1,56)=4.91; p <0.05] inhibited Na^+^,K^+^-ATPase activity but LPS did not potentiate this effect. Statistical analysis (three-way ANOVA) revealed that NAC administration attenuated the decrease Na^+^,K^+^-ATPase activity [significant drug (Saline or GA) by toxin (Saline or LPS) by treatment (Sal or NAC) interaction: [*F*(1,56)=4.07; p <0.05] induced by GA and LPS (Figure 7A).

We also investigated whether GA and LPS could inhibit selectively some isoform of Na^+^,K^+^-ATPase. For this purpose we used a classical pharmacological approach based on the isoform-speciﬁc sensitivity to ouabain [45]. Statistical analysis (three-way ANOVA) revealed that NAC administration protected against the decrease Na^+^,K^+^-ATPase (α1-subunit) activity [significant drug (Saline or GA) by toxin (Saline or LPS) by treatment (Sal or NAC) interaction: [*F*(1,56)=4.50; p <0.001] induced by GA and LPS (Figure 7B).

### Effect of treatments on Hippocampal volume

**Figure 8:**
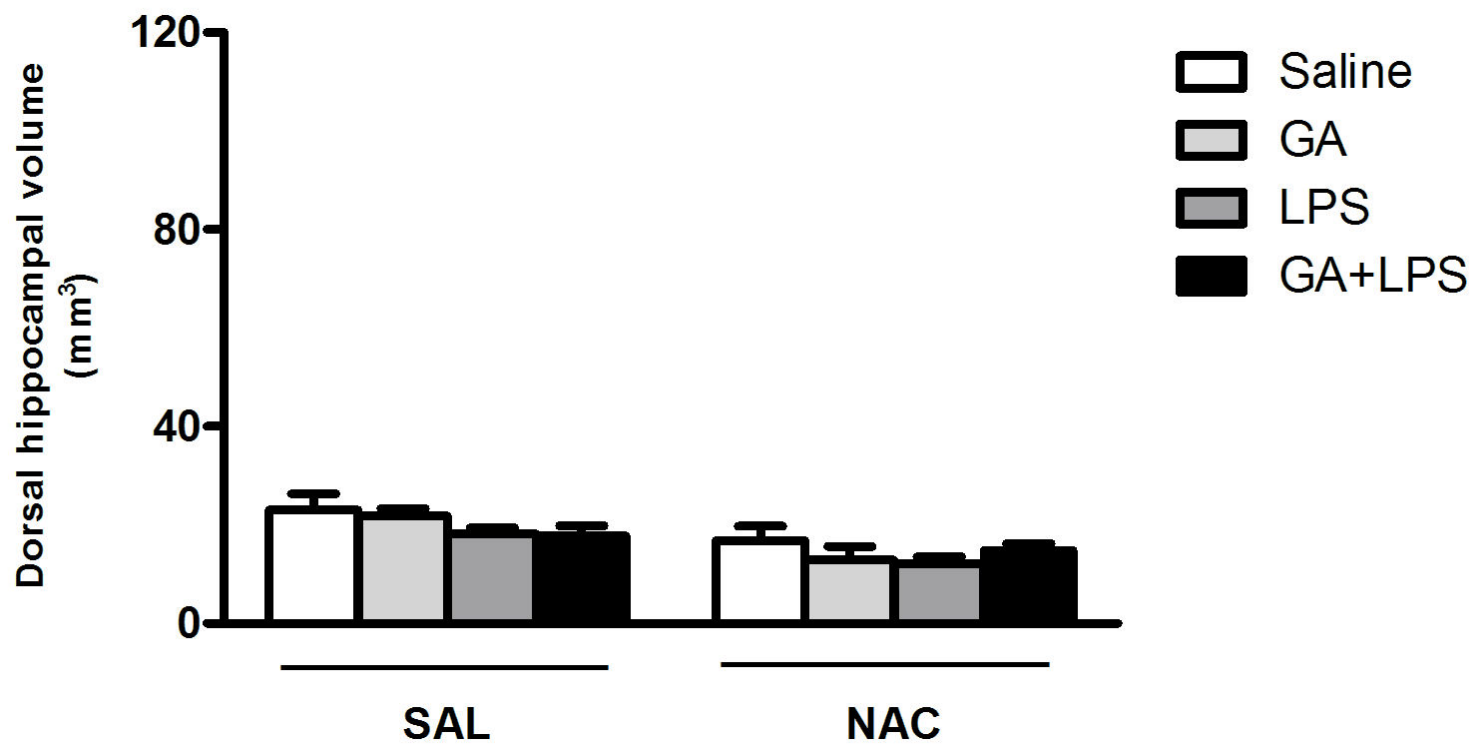
Effect of early postnatal chronic GA, NAC and LPS administration on hippocampal volume. Data represent means ± S.E.M. for n = 5-6 in each group. No significant differences between groups were detected.

**Figure 9:**
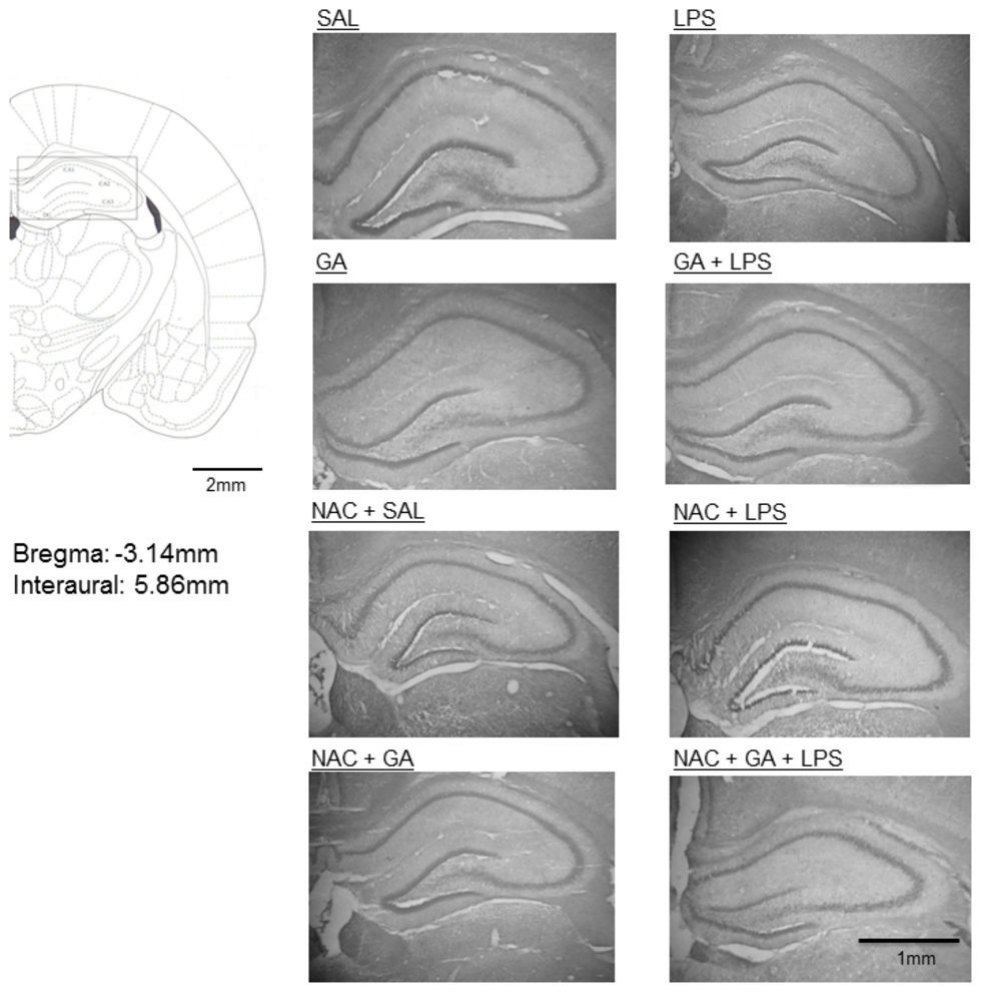
Digitized images of dorsal hippocampus showing similarities in the volume of this region between the different experimental groups. Schematic drawing obtained from Paxinos and Watson's Atlas.

Figure 8 shows that there was no difference between groups concerning hippocampal volume of rat pups. Statistical analysis of hippocampal volume (three-way ANOVA) did not show a significant drug (Saline or GA) by toxin (Saline or LPS) by treatment (Sal or NAC) interaction: [F(1,38)= 0.08; p> 0.05]. Figure 9 shows a representative figure of dorsal hippocampus of all treatments.

## Discussion

Patients with GA-I usually present acute clinical features early in life resulting from metabolic decompensation with respiratory distress and neurological symptoms, including psychomotor delay, irritability, lethargy, hypotonia, convulsions, and coma. Most children survive to the first acute metabolic crisis, but develop long-term complications including neurological [5-7]. Although it is believed that these abnormalities occur as a result of the primary metabolic impairment, the underlying mechanism of brain damage and neurological deficits in GA-I is poorly understood.

It is known that patients and experimental models of this IEM exhibit neuronal damage and changes in several areas of the central nervous system, as well as in several other neurological diseases [8,10,12,13]. This is often related to oxidative stress and neuronal death, causing cognitive impairment and neuroinflammatory processes [9-13-19-22]. A recent study in an experimental model has elucidated the role of inflammatory mediators in model of acute seizures by GA [11]. However, it is not known if GA itself may increase these mediators and if they are related with memory deficit.

This current study was carried out with young rats in a period of development with proven synaptogenesis and cell proliferation in several brain structures involved in learning and memory. We found that the chronic GA administration (5^th^ - 28^th^ day of life) at doses that raise its concentration in blood and in the brain [29] and LPS injection cause memory deficits in spatial recognition, decrease antioxidant defenses, and induce increased levels of pro-inflammatory cytokines (IL-1β and TNF-α) in the hippocampus. In addition, an increasing oxidative markers and consequent inhibition of the Na^+^, K^+^-ATPase activity, without modifying hippocampal volume was observed.

It is important to observe that chronic GA, LPS or NAC administration had no effect on body weight, implying that chronic injection did not cause malnutrition in animals (Figure 1). Similarly, the same treatments did not change rat performance in the open field task as observed by a number of rearing and crossing at the testing session (Figure 2). In addition, these treatments did not change the behavior of rat pups in the elevated plus maze task, indicating that GA, LPS and NAC were not anxiogenic (Table 1).

As regards to the behavior of rat pups, we observed that LPS potentiated the deficit of spatial learning induced by GA (Figure 3 A-C). This result suggests that early chronic postnatal administration of GA caused a long-standing deficit in spatial learning probably secondary to GA-induced brain dysfunction [29], which was potentiated by an inflammatory insult. In fact, studies have suggested that neonatal inflammation causes cognitive impairment in humans and experimental models [53-55]. Taken together, we can suggest that the potentiation of cognitive deficit induced by GA neonatal administration after inflammatory insult could be involved with the cognitive dysfunction presented in GA-I children [5-7], since these patients frequently develop neurological dysfunction after an infectious process [50-56].

In line of this view, our data revealed that spatial learning deficit induced by GA and LPS was associated with an increase of proinflammatory cytokine levels such as TNF-α and IL-1β (Figure 4A and B), and that the co-administration of these compounds potentiated IL-1β levels in the hippocampus of rat pups (Figure 4 B). It is important to note that the cytokines such as IL-1β and TNF-α are primarily synthesized and released by glial cells that can be activated by trauma, infection or the presence of endogenous yet abnormal protein aggregates [57,58]. Since the activation of microglia produces a variety of inflammatory mediators [59] and the exposure to GA activates glial cells [22], it is plausible to propose that the increase of cytokine levels induced by GA may be due to glial activation and that LPS potentiated this effect manly by increasing IL-1β levels (Figure 4B).

Current evidence indicates that cytokines, particularly IL-1β, increase neuronal excitability by activating IL-1 receptors [64]. The neuronal IL-1R1 stimulation induces Src kinase-mediated tyrosine phosphorylation of the NR2B subunit in N-methyl-D-aspartate (NMDA) receptor. As a consequence, IL-1β facilitates NMDA receptor-mediated Ca^2+^ influx into neurons, promoting excitotoxicity [102]. Considering that IL-1β can also inhibit glutamate uptake in astrocytes [67] and increase its glial release possibly via TNF-α production [94], the increase of pro-inflammatory cytokines may result in elevated extracellular glutamate levels and toxicity in this model of organic acidemia. In agreement of this view, a considerable body of evidence has demonstrated that glutamate receptor modulation can lead to GA-induced toxicity [9,11,20,21]. Furthermore, the administration of LPS can also impair memory and elevate hippocampal IL-1β levels [64] by increasing the GABAergic inhibition [65,66].

Since synaptic neurotransmission changes induced by cytokine may result in memory impairment [66,67], the actions of GA and LPS in neurotransmission [64] could potentiate the cognitive deficit observed in this study. In agreement to this, it has been observed in post mortem brain of GA-I patients pronounced reactive hypertrophic astrocytes with chronic astroglyosis and reactive microglia [60-63].

Furthermore, TNF-α and IL-1β act in their respective receptors and cause activation of nuclear factor kappa-light-chain-enhancer of activated B cells (NFκB), a transcription factor that migrates to the cell nucleus and can promote elevated concentrations of intracellular calcium as well as a production of NO and H_2_O_2_ increase [95,96]. NO is a free radical that when reacting with superoxide anion (O_2_^•-^, also formed during the inflammatory process by enzymes such as NADPH oxidase) generates the highly reactive peroxynitrite (ONOO^-^) [97]. These free radicals induce a nitrosative/oxidative stress that may result in DNA damage, increase of lipid peroxidation and impaired antioxidant defenses [71,72].

Interestingly, we revealed that GA and LPS administration increased TNF-α and IL-1β levels as well as increased lipid peroxidation and impaired antioxidant defenses in the hippocampus of rat pups. Regarding the antioxidant defenses, the concentrations of GSH, which is the major brain antioxidant, were reduced in the hippocampus of the animals that received GA and LPS. Considering that GSH is an effective scavenger of free radicals [68] and that a disruption of GSH system homeostasis by neuroinflammation [97] may result in oxidative injury in sensitized neurons [69,70], reduced GSH levels in hippocampus caused by GA and LPS administration may be at least in part involved in the memory deficit observed in these rat pups. Furthermore, GA and LPS decreased CAT and SOD activities in the hippocampus (Figure 6C-D), suggesting that the reduction of CAT and SOD activities did not present a compensatory mechanism in response to the increased formation of reactive species. In fact, oxidative stress accompanies inflammatory processes [73] and patients with GA-I frequently develop cognitive damage during and after infections associated with inflammation and with a potential increase of tissue concentrations of GA [52,74]. Also, reactive astrogliosis, a characteristic feature of neuroinflammation, is often found in brain GA-I patients [60-63].

Considering that GA, inflammatory mediators and oxidative damage have been implicated as negative modulators of Na^+^, K^+^-ATPase [9,20,75-77], which is the major determinant of sodium concentration and cellular excitability, we investigated if the mediators could alter the enzyme activity. In fact, studies showed that proinflammatory cytokines such as IL-1β and reactive species have been shown to decrease the expression of Na^+^, K^+^-ATPase during severe experimental sepsis [78].

In our investigation it was interesting to learn that besides LPS and GA have increased oxidative and inflammatory markers, these compounds inhibited markedly Na^+^, K^+^-ATPase activity suggesting the participation of this enzyme in spatial memory deficit in this acidemia model. These results are in accordance with Liu and colleagues [79] that showed an inhibition of the activity of this enzyme by LPS. In addition, Lima et al. [80] showed that in situations which cause brain inflammation and oxidative stress such as traumatic brain injury, the inhibition Na^+^, K^+^-ATPase activity occurs concomitantly to a cognitive impairment. Some studies have also shown that the inhibition of Na^+^, K^+^-ATPase activity induces spatial learning deﬁcits [80,81] and impairs retention of an inhibitory avoidance task in rats [82]. In fact, Na^+^, K^+^-ATPase activity can be inhibited by the direct and indirect action of LPS [79,83,84], as well as by GA and LPS-induced free radical and inflammatory mediator generation [85,86]. Thus, it is plausible to propose that some of the mechanisms may result in the Na^+^, K^+^-ATPase inhibition and contribute with memory impairment observed in this study.

Although we have shown that GA and LPS facilitate the memory deficit, in the present study we did not find changes in the dorsal hippocampal volume after GA, LPS or GA/LPS administration (Figures 8 and 9). These results agree with studies that show the GA-I patients develop mainly bilateral striatal degeneration, but not in hippocampus, during catabolic or infectious events observed in neuroradiological imaging [62,87-89]. In fact, Oliveira-Bravo et al. [77,90] showed that a single intracerebroventricular dose of GA induced degeneration mainly in the striatum. Furthermore, Ulrich et al. [91] showed that only 3-OH-glutaric, but not GA and glutaconic acid induced neurodegeneration in corticostriatal and hippocampal slice cultures from rat brain. Thus, this result reveals that GA did not induce reduction of hippocampal volume, suggesting that the memory deficit in rat pups was not due to tissue lesion in this GA-I model.

Since we observed the increase of inflammatory and oxidative marker levels in the hippocampus of rat pups after the GA and LPS administration, we decided to test whether the chronic co-treatment with NAC, an agent with antioxidant and anti-inflammatory properties, could protect the alterations induced by GA and LPS. In fact, the results presented in this report showed that NAC supplementation was effective in preventing the decreased cognitive deficit as well as the increase of inflammatory and oxidative mediators levels induced by GA and LPS in rat pups. The supplementation of this compound also protected against the Na^+^,K^+^-ATPase activity inhibition (total and α1 subunit). 

Recent studies have shown that cytokines have potential to alter the redox equilibrium, thereby affecting GSH/GSSG shuttling and recycling [97]. For example, after H_2_O_2_ or amyloid-β intracerebral injection in rat brain, it was observed an association between lipid peroxidation and the levels of cytokines in addition to a significant inverse correlation between glutathione peroxidase activity and lipid peroxidation levels [98,99]. In this line of view, we showed that the treatment of rat primary astrocytes with TNF-α or IL-1β leads to marked alteration in cellular redox (decrease in intracellular GSH). On the other hand, pretreatment of astrocytes with NAC, an antioxidant and efficient thiol source for glutathione, prevents cytokine-induced decrease in GSH [100,101]. In fact, our findings showed that NAC supplementation decreased cytokine levels and TBARS production as well as increased GSH content and improved memory deficit induced by GA and LPS. Accordingly, glutathione prevented Na^+^,K^+^-ATPase activity inhibition GA-induced in neuronal cultures [92]. These data suggest that inflammatory events and oxidative stress occur in a synchronized manner by changing the redox environment, and that compounds with antinflammatory and antioxidant properties could be used as adjuvant therapy to some neurodegenerative disorders. 

Thus, a currently reported increase in GA-induced spatial memory deficit by LPS not only supports the idea that inflammatory mediators, such as IL-1β facilitates the cognitive impairment, but also adds pharmacologic evidence to the fact that infections and oxidative damage precipitate metabolic crises and worsen neurologic status of patients with GA-I. Furthermore, the Na^+^, K^+^-ATPase activity inhibition also plays an important role for the cognitive impairment observed in this work. Taken together, all experimental findings suggest that oxidative stress GA and LPS-induced impairs the intrinsic cell potential, leading to proinflammatory signals and creating a vicious circle between oxidative stress and neuroinflammation causing a negative modulation in Na^+^,K^+^-ATPase activity that was responsive to NAC supplementation. Therefore, if the effects detected in this study also occur in patients with GA-I, it is tempting to propose that they may contribute, at least in part, to the neurological dysfunction found in GA-I, and that the administration of NAC could represent a complementary therapy together with Lys dietary restriction and L-carnitine supplementation in the treatment of children with glutaric acidemia.
